# Experimental Detection of Preferred Lanthanum Siting in Zeolite Y and Its Impact on Catalyst Reactivity

**DOI:** 10.1021/acs.jpcc.5c00474

**Published:** 2025-04-23

**Authors:** Anya Zornes, Omio Rani Das, Nabihan B. Abdul Rahman, Jacob Crouch, Steven Crossley, Bin Wang, Walter Alvarez, Matthew J. Wulfers, Daniel E. Resasco, Jeffery L. White

**Affiliations:** † School of Chemical Engineering, Oklahoma State University, 420 Engineering North, Stillwater, Oklahoma 74078, United States; ‡ School of Sustainable Chemical, Biological, and Materials Engineering, University of Oklahoma, Norman, Oklahoma 73019, United States; § Phillips 66, Bartlesville Technology Center, Bartlesville, Oklahoma 74004, United States

## Abstract

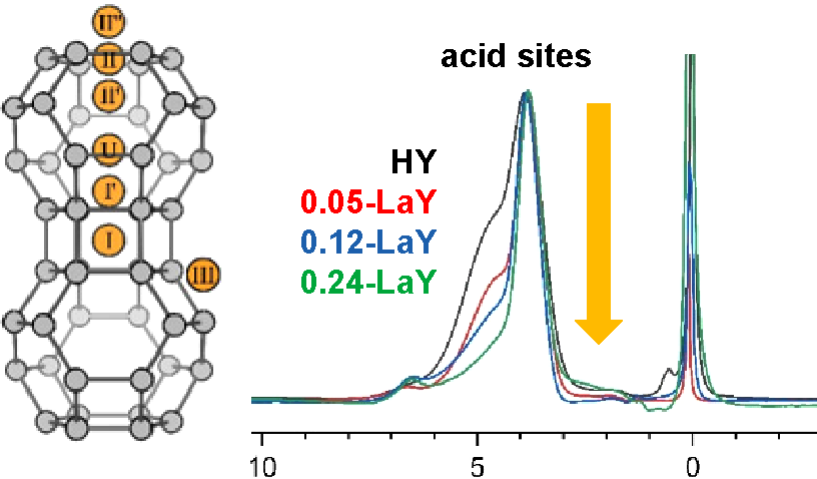

Lanthanum and other rare earth cations are routinely added to commercial zeolite catalysts to increase stability during hydrothermal regeneration steps, as well as to modify catalyst reactivity by changing the distribution of acid sites and operative electric field gradients in working catalysts. Solution ion-exchange procedures are typically used to introduce La cations into zeolite Y, primarily as La^3+^ or its hydroxylated moieties, La^3-n^(OH)_n_, in the formulation of commercial LaY or ultrastable steam-stabilized Y (USY) catalysts. Within zeolite Y, multiple possible exchange sites exist for La occupation, but quantitative measurement of La siting as a function of La loading in the catalyst is not generally accessible. Specific open questions involve whether La cations reside in both sodalite and supercage sites, which sites are preferred for La incorporation, and whether La can be selectively incorporated into specific site types. In this contribution, a simple quantitative method based on solid-state NMR coupled with the preparation of La–HY catalysts without framework defects reveals that at low La loadings of less than 3 wt %, essentially all La is incorporated into sodalite cages as La^3+^ ions. DFT calculations support these experimental conclusions. Coincident with this incorporation, sodalite Brønsted bridging acid sites (BAS) decrease, but the number of supercage BASs can remain constant depending on the La concentration. Increased La loadings in the catalyst preferentially reduce the number of sodalite BASs compared to supercage acid sites, with both sodalite and supercage BAS amounts, as well as the amount of newly created La^3-n^(OH)_n_ species, quantitatively measured using the methods described here. Flow reactor hexane-cracking experiments, as well as in situ probe reactions, reveal that catalyst reactivity increases relative to that of HY when La resides exclusively in sodalite positions.

## Introduction

Zeolites have been successfully employed as industrial cracking, reforming, hydrogen-transfer, and hydrocarbon synthesis catalysts for decades.^1−4^ Requirements for increased selectivity to minimize the production of deleterious molecules from conventional hydrocarbon feeds, increased conversion from nonconventional feedstocks like waste and biomass, and reduced operating costs afforded by longer catalyst lifetimes necessitate continuous improvements in understanding the structure and activity relationships of these dynamic and compositionally heterogeneous silicoaluminate materials.^5−8^ The faujasite class of zeolites, most notably zeolite Y, is used in fluid catalytic cracking (FCC) of gas-phase hydrocarbons at high temperatures typically following postsynthetic metal cation exchanges into the zeolite interstitial spaces to increase catalyst performance and lifetime. Zeolite HY, into which relatively low amounts of rare earth (RE) cations like lanthanum and cerium are exchanged, is known to exhibit increased hydrothermal stability in the presence of gas-phase water during steam-regeneration steps.^9−12^ However, with the increased need to employ some large-pore zeolites like HY to convert oxygenated feedstocks that evolve water during reactions, often at lower temperatures where liquid water can be present instead of water vapor,^13,14^ coupled with increased demands on overall carbon maintenance in traditional chemistries, requires a more detailed understanding of how rare earths like La impact the structure and reactivity of zeolite HY.

Specific outstanding questions regarding La incorporation in zeolite Y that could be addressed by improved experimental methods include: (1) Does La have a preferred site for incorporation in zeolite Y?; (2) Where does La reside in the catalyst structure as a function of the amount of La incorporated, i.e., the La-to-framework Al ratio (La/Al), and how does this impact the number of Brønsted acid sites in sodalite vs supercage positions?; (3) What is the state of the incorporated La cation as a function of La loading and catalyst preparation method, e.g., La^3+^ vs La^3–n^(OH)_n_?; (4) How do the results of (1)–(3) impact La-HY reactivity? These questions are not new, and previous computational and experimental studies have attempted to address them at various levels. For example, cation locations have been inferred via assessment of unit cell sizes measured via X-ray diffraction,^15−18^ in several spectroscopic studies primarily involving infrared or NMR methods,^10,11,19−37^ and in aberration-corrected microscopy studies.^38^ Common to many of these studies is the fact that the standard aqueous ion-exchange and heating methods used to introduce La cations cause hydrolysis of the zeolite Y framework prior to complete La incorporation, further complicating detection and interpretation of preferred La siting. Recently, it has been demonstrated that even room-temperature aqueous exchange and water removal methods, which are significantly milder than those typically employed for La introduction, degrade the zeolite Y framework, preferentially inducing hydrolysis of sodalite cage acid sites relative to supercage sites.^39^

In this contribution, we demonstrate that La-exchanged HY with different La content can be prepared without introducing framework defects into the catalyst, and introduce a ^1^H MAS standard-addition NMR experimental strategy that quantitatively and selectively measures the impact of La introduction in sodalite vs supercage sites. These quantitative data guide the interpretation of the impacts of La incorporation on high-temperature flow-reactor hexane cracking data as well as room-temperature in situ isotopic exchange experiments with toluene-d_8_, and provide a quantitative basis for comparing LaHY to the initial HY catalysts. Key results of this study demonstrate that: (1) LaHY catalysts can be prepared with no silanol defects or framework hydrolysis; (2) La^3+^ cations can be selectively incorporated into *only* sodalite cage positions and preserved there without further hydrolysis to lanthanum hydroxides, with experimental results supported by computational calculations; (3) catalysts with La^3+^ exclusively residing in sodalite cages exhibit the largest reaction rates per unit La for both high-temperature hexane cracking and room-temperature H/D exchange, corresponding to ca. one La ion per 25 Al atoms; and (4) increasing La loading beyond ca. 1 La ion per 10 Al atoms corresponds to the formation of La^3–n^(OH)_n_ species and concomitant decreases in reaction rates for the aforementioned reactions. In total, these findings indicate not only the preferred siting of La in sodalite cage positions of HY but also the active role of both proton and La sites in the sodalite cages, even for molecules that are ostensibly too large to access them. Further, the data provide quantitative guidance for determining the optimum La amounts to employ in LaHY catalysts.

## Experimental Section

### Catalyst Samples

Zeolite NH_4_Y with a nominal Si:Al ratio of 2.6 was obtained from Zeolyst International. HY was prepared from NH_4_Y in a glass reactor by stepwise vacuum dehydration, with a pressure of less than 1 × 10^–4^ Torr maintained throughout the process, heating at 0.5 °C/min to 100 °C, holding for 2 h, heating at 2 °C/min to 450 °C, and holding for 12 h. La–Y was prepared from NH_4_Y by exchanging 500 mg NH_4_Y in either 20 mL (higher three loadings) or 10 mL (lower three loadings) of aqueous La(NO_3_)_3_ solutions of appropriate concentration for 3 h at 90 °C. Samples were vacuum filtered, rinsed with DI water, and then dried in a vacuum oven at 80 °C overnight. The samples were then fully dehydrated by stepwise vacuum dehydration, with pressure maintained at less than 1 × 10^–4^ Torr throughout, heating at 0.5 °C/min to 350 °C, holding for 10 min, then heating at 2 °C/min to 550 °C, and holding for 2 h. The low heating rate for LaY was continued to a higher temperature than when preparing HY, as both deammoniation and the removal of physically adsorbed water in La-exchanged zeolite Y occur at 350 °C,^40^ and it is undetermined how well La^3+^ protects the framework at these lower temperatures, since the hydration enthalpy of LaY is more similar to HY than to other cation-exchanged zeolites.^41^ The LaY samples were also heated to a higher final temperature than HY, as the La(OH)_n_ complexes make it difficult to fully remove water, and the majority of dehydroxylation does not occur until around 500 °C.^36,39−42^Table 1 summarizes the samples and naming convention used throughout the study, including results from the elemental analysis of each prepared sample. Note that the elemental La/Al values in the last column of Table 1 indicate an upper limit of incorporated La, irrespective of the synthesis solution concentration.

**Table 1:** La–Y Naming Conventions, Synthesis Solution Concentrations, and Elemental La:Al Ratios in the Final Catalysts

Sample	La(NO_3_)_3_ conc.	Solid:liquid ratio	La/Al (soln.)	La wt% (cat.)	La/Al_f_ (cat.)
0.05-LaY	0.01 M	1g:20 mL	0.05	2.82	0.04
0.12-LaY	0.025 M	1g:20 mL	0.12	6.65	0.11
0.24-LaY	0.05 M	1g:20 mL	0.24	9.34	0.16
0.97-LaY	0.1 M	1g:40 mL	0.97	10.0	0.17
2.4-LaY	0.25 M	1g:40 mL	2.4	9.95	0.17
4.8-LaY	0.5 M	1g:40 mL	4.8	--	0.17

**Figure 1:**
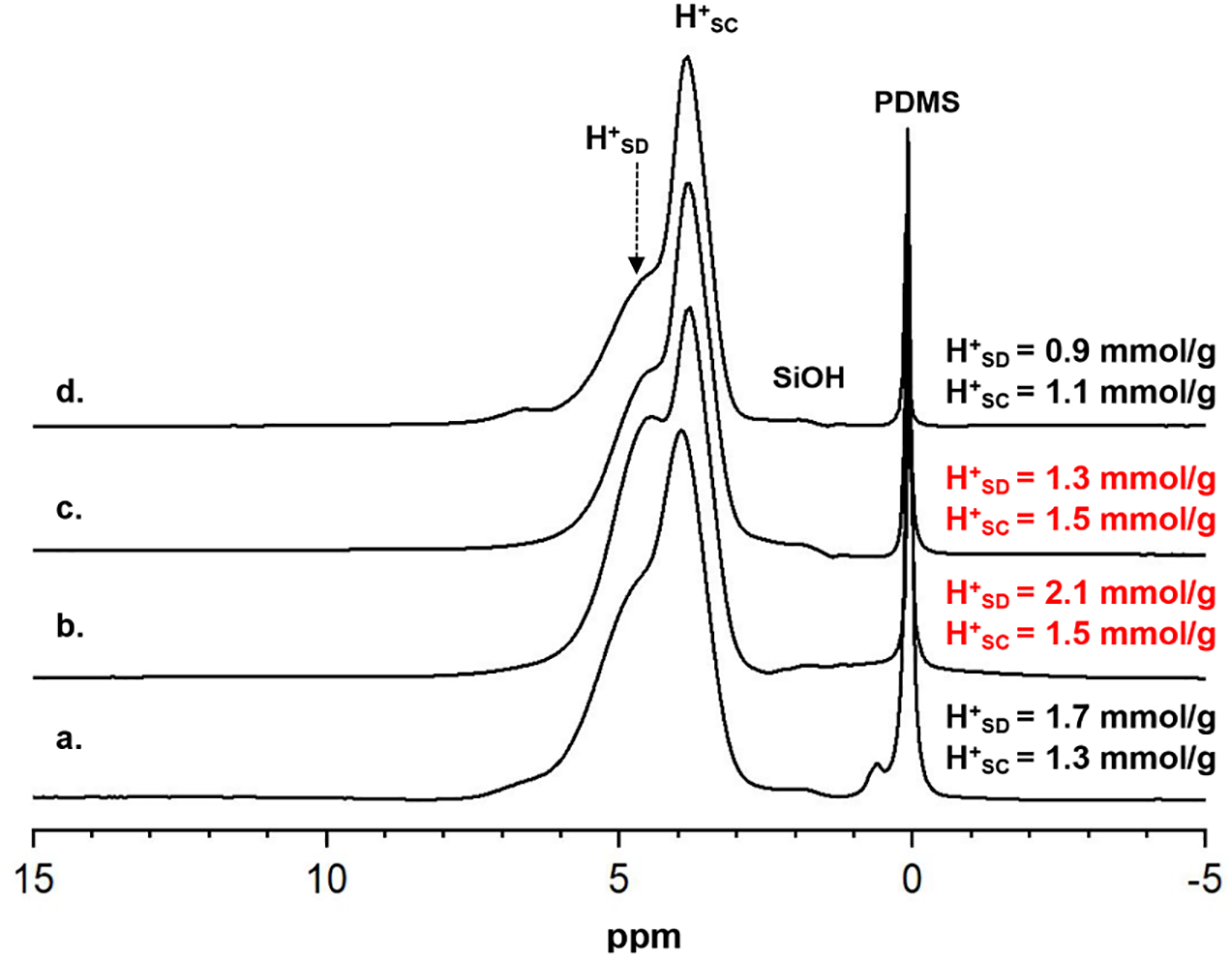
^1^H MAS NMR spectra of the (a) HY catalyst prepared from the parent NH_4_Y via low ramp-rate vacuum dehydration; (b) same catalyst as in (a) but following a three-time NH_4_Cl exchange prior to dehydration; (c) same catalyst as in (b) followed by exchange with 0.01 M La(NO_3_)_3_ prior to dehydration; (d) same catalyst as in (a) followed by exchange with 0.01 M La(NO_3_)_3_ prior to dehydration. The absolute number of BAS protons in the sodalite cages (signal at 4.9 ppm) and in the supercages (signal at 3.9 ppm) are reported as **H**^**+**^_**SD**_ and **H**^**+**^_**SC**_ on the right of each spectrum as obtained from a two-component deconvolution following the procedure in Figure S1 and as previously published.^39^ Note the absence of any SiOH signals. PDMS denotes the internal standard polydimethylsiloxane.

To further eliminate any contributions from residual Na ions that are present in the commercial NH_4_Y starting material, several control experiments were done in which three additional ion-exchange steps with NH_4_Cl prior to the generation of the HY or LaY forms, were executed, as discussed in Figures 1 and 6 below.

Some HY and LaY samples were then exposed to ambient air at 24 °C with a relative humidity of 30–45% in shallow bed conditions (bed height of less than 1 mm) for 1 week. The samples were then dehydrated again in the glass reactor at pressures of less than 1 × 10^–4^ Torr with the same heating procedure was used to make HY. It is known that HY will undergo dealumination from exposure to ambient air and that heating such a sample will further destroy the framework.^43−45^Table 2 summarizes the naming conventions for samples before and after exposure to ambient moisture. Any sample denoted as LaY still contains significant BASs and is actually a LaHY catalyst but denoted as the former for convenience.

**Table 2:** Naming Conventions vs Sample History

Notation	Sample history
NH_4_Y	Commercial CBV300 as received
HY	CBV300 calcined (deammoniated and dehydrated) in vacuum
X-LaY	NH_4_Y exchanged with La(NO_3_)_3_ then heated to 550 °C
Y-1wk	HY or X-LaY exposed to ambient air for 1 week, then reheated to 450 °C

### NMR Hardware and Sample Packing

All NMR experiments on the solid catalysts were conducted at 9.4 T with a Bruker Avance II console using a 4 mm double-resonance MAS probe. Samples were packed in ZrO_2_ rotors, under either ambient air (^27^Al and ^29^Si NMR) or in an inert argon gas atmosphere by using a VAC atmosphere glovebox (^1^H NMR).

### ^1^H NMR Experiments

^1^H NMR spectra were acquired on fully dehydrated catalysts using a single 90° excitation pulse of 3.67 μs. In all cases, 64 transients were obtained with a recycle delay of 60 s, which is in excess of 5 times the longest relaxation time observed at a spinning speed of 10 kHz. Pulse durations and chemical shifts were calibrated using hexamethylbenzene.

For quantitative spin-counting experiments, measured amounts of vacuum-dehydrated sample and an inert quantitation standard, poly(dimethylsiloxane) (PDMS), were packed in the middle third of a ZrO_2_ rotor, between sulfur (bottom) and a Teflon spacer (top).

### ^27^Al NMR Experiments

^27^Al NMR spectra were acquired on ambient-air hydrated samples with a 15° pulse of 0.92 μs and a recycle delay of 0.2 s for 4096 transients at a spinning speed of 10 kHz. Pulse durations and chemical shifts were calibrated by using a 0.1 M aqueous solution of Al(NO_3_)_3_.

### ^29^Si NMR Experiments

^29^Si NMR spectra were acquired on ambient-air-hydrated samples with a 90° pulse of 4.3 μs. All spectra were acquired for 1024 transients with a recycle delay of 60 s and a spinning speed of 10 kHz. Pulse durations and chemical shifts were calibrated by using PDMS.

### XRD

Powder X-ray diffraction data were acquired at ambient humidity using a Philips X-ray diffractometer (Phillips PW 3710 MPD, PW2233/20 X-ray tube, copper tube detector, wavelength 1.5418 Å), which operated at 45 kW and 40 mA. Diffractograms were obtained with 2θ ranging from 2° to 45° and with a diffractometer difference of 0.02°.

### Elemental Analysis

Elemental analysis to determine the weight percent (wt%) of lanthanum in the exchanged samples was provided by Galbraith Laboratories using the GLI ME-70 procedure.

### Kinetic Measurements

The impact of lanthanum loading on catalyst activity was measured using two model reactions: hexane cracking in a continuous-flow microreactor and in situ H/D exchange solid-state NMR experiments using toluene-d_8_ as the reagent. For hexane cracking reactions, catalyst samples were pelletized to a size of 250–425 μm with a mass of 50 mg. Prior to the reaction, samples were pretreated to remove any moisture present by heating to 120 °C at 0.5°/min, holding at 120 °C for 2 h, heating to 300 °C at 2°/min, and holding at 300 °C for 1 h. Following this dehydration pretreatment, catalysts were heated to the reaction temperature of 425 °C at 2°/min. Both the reaction and pretreatment were conducted under a 100 mL/min flow rate of nitrogen carrier gas, to which liquid hexane was injected at a rate of 0.5 mL/h after reaching the reaction temperature. Each run was conducted for approximately 6 h, with analysis of reaction products carried out using an online GC with an FID detector and an HP-Plot Al_2_O_3_ column.

In-situ H/D exchange experiments were carried out at room temperature using catalyst samples loosely packed in a ZrO_2_ rotor in an inert argon atmosphere, which were then evacuated under vacuum. Toluene-d_8_ was adsorbed onto the catalyst such that, for all samples, a 1:1 molar ratio of reactant to acid sites and (when applicable) lanthanols was achieved. The rotor was then sealed using a Teflon spacer and kept in a liquid nitrogen bath until the sealed rotor was inserted into the NMR probe. ^1^H NMR spectra were then obtained at room temperature over the course of the reaction, with the integrated area of each peak correlating directly with the concentration of that species. Rate constants for the reaction were determined using a least-squares fit of the growth of the toluene peak area T(t) during the short-time initial-rate region (≤20 min) of the proton/deuterium exchange curve using the following equation:
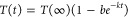


where fully deuterated toluene is assumed to be in excess under initial conditions. Corrections for adsorption equilibria or diffusion limitations were not necessary, as toluene was fully adsorbed prior to proton exchange.^46,47^ Catalyst sample preparation and reactant adsorption were spatially removed from the location where the in situ NMR experiments are completed. Immediately following toluene adsorption, the sample was immersed in liquid nitrogen for transport and kept cold until placed into the spectrometer a few minutes later. While every effort was made to preclude any exchange reaction prior to acquiring the first spectrum, invariably some H/D exchange had already occurred during the several-minute delay, with more occurring as the catalyst reactivity increases thus contributing to larger deviations from the origin for the most active catalysts in Figure 6 below (vide infra).

### Computational Methods

All density functional theory (DFT) calculations were performed using the Vienna ab initio simulation package (VASP)^48^ with projector augmented wave (PAW) potentials^49^ and the Perdew–Burke–Ernzerhof (PBE) exchange and correlation functional.^50^ The structural relaxation was carried out with a kinetic cutoff energy of 400 eV. All atoms were fully relaxed until the atomic net force was less than 0.02 eV Å^–1^. The DFT-D3 method^51^ was used to include van der Waals interactions. The KPOINTS was set to 3 × 3 × 3 to sample the Brillouin zone.

A rhombohedral primitive cell was used (with 48 T sites)^52^ and the unit cell was relaxed, allowing it to expand following the addition of La species. To match the experimentally determined Si/Al ratio of 3, 12 Al atoms were placed in the unit cell, with the specific distribution determined according to Loewenstein’s rule^53^ and comparison of calculated ^29^Si NMR spectra to experimental data. The formation energetics of a LaY structure at each of the 7 unique cation locations was determined by the following equation:
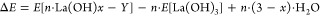
where *E*[*n*·La(OH)*x*–*Y*], *E*[La(OH)_3_], and *E*[H_2_O] are the DFT-calculated total energies of Y zeolite with La species incorporated, a La(OH)_3_ cluster, and a water molecule. The value of *n* equals 1–3 in the calculations to represent the La monomer, dimer, and trimer. It should be emphasized that the exact value of this formation energy varies when the references are changed, but the trend and relative stability remain consistent.

## Results and Discussion

### Measuring the Impact of La on Sodalite vs Supercage Acid Sites

Dry samples of the initial commercial HY catalyst, as well as the prepared 0.05-to-4.8 LaY catalysts detailed in Table 1 were examined using a quantitative ^1^H spin-counting or standard-addition MAS NMR method employing known amounts of inert polydimethylsiloxane (PDMS) as an internal calibrant to yield absolute amounts (mmol/g) of each type of proton in the catalyst. As demonstrated in several prior studies of zeolite catalysts, the method is quantitative with respect to different BAS protons and silanol (SiOH) concentrations, with standard deviations of 0.05–0.1 mmol/g for typical zeolites and 0.01–0.05 mmol/g for control experiments on known calibrants.^39,54,55^ Indeed, recently reported standard deviations of 0.5–0.7 for commercial HY with Si/Al = 2.6 were much larger than prior zeolite or silicoaluminophosphate studies, which indicated a deficiency in the overall stability of HY under standard catalyst preparation conditions and led to improved preparation protocols.^39^ Here, La was introduced to the significantly more stable NH_4_Y rather than NaY or HY and then dehydrated with low ramp-rate heating under vacuum, rather than the standard practice of dehydrating under ambient air or flowing air. Further, prior to the acquisition of any spectroscopic data probing La siting, the La-exchanged catalysts were not exposed to ambient moisture.

For reference and convenience to the reader, Figure 1 shows the quantitative ^1^H MAS NMR results for an important control experiment involving the commercial HY catalyst used as the parent material for all LaHY samples. In the HY (1a, 1b) and LaY (1c, 1d) spectra, only three peaks are observed, including the PDMS spin-counting standard near 0 ppm, the supercage BAS hydroxyl protons (BAS-sc) at 3.9 ppm, and the sodalite BAS hydroxyl protons (BAS-sd) at 4.9 ppm. Importantly, there are no peaks observed in the 1.5–2.5 ppm region, as typically reported for HY catalysts,^22^ even for the LaY catalysts in parts 1c and 1d, indicating that internal silanol groups resulting from framework hydrolysis are absent. The absence of measurable SiOH or AlOH signals in the 1.5–2.5 ppm region indicates that dry, high-Al-content HY and La–HY samples can be prepared without introducing framework defects, as previously demonstrated for HY zeolites.^39^ Using the simple deconvolution procedure shown in Figure S1, the amount of supercage and sodalite unit acid sites can be quantified and is reported to the right of each spectrum in Figure 1.

The progression of sodalite vs supercage acid site signals in Figure 1a–d, denoted H^+^_SD_ and H^+^_SC_ respectively, provides clear experimental evidence that La can be selectively incorporated into sodalite sites while preserving the integrity of the framework, including the sodalite units themselves. Figure 1a shows the parent HY prepared from the commercial NH_4_Y material, with relatively more sodalite acid sites than the supercage and a total [BAS] = 3.0 mmol/g, well below the theoretical value of ca. 4.2 mmol/g based on the Si/Al ratio. Following additional exchange with NH_4_Cl solutions, Figure 1b shows that the total [BAS] increases to 3.6 mmol/g, with the majority of that increase taking place due to the [H^+^_SD_] change from 1.7 to 2.1 mmol/g, which is relatively larger than the increase observed for [H^+^_SC_]. The additional ammonium-exchange steps from Figure 1a,b displace residual Na^+^ cations remaining from the synthesis,^56^ which, according to the increase in sodalite vs supercage acid sites, preferentially remain in sodalite cages. The HY catalyst in Figure 1b, having a [BAS] much closer to the theoretical limit with residual Na^+^ removed and without any framework defects based on the absence of SiOH signals, is now an ideal catalyst to use for introducing small amounts of La. The spectrum in Figure 1c is for the same catalyst as used to make the HY shown in Figure 1b, but after exchange with 0.01 M La(NO_3_)_3_ solutions prior to dehydration. The catalyst in Figure 1c is equivalent in its preparation to the 0.05-LaY listed in Table 1. The red text in the right captions of Figure 1b,c highlights that upon La incorporation, the *only* signal that is impacted is that for the H^+^_SD_ sites. The [H^+^_SD_] decreases from 2.1 to 1.3 mmol/g upon La incorporation, while the [H^+^_SC_] remains constant at 1.5 mmol/g, and again no framework defects are created. Even without doing the extra ammonium exchange steps on the parent Y catalyst to remove residual Na^+^, i.e., starting with the catalyst in Figure 1a, the same La-incorporation procedure incorporates ca. 80% of all the La cations into the sodalite units, as can be seen by comparing the data in Figure 1d to Figure 1a. The LaY catalyst in Figure 1d, following exchange from the catalyst in Figure 1a, has slightly more than half of the initial [H^+^_SD_] while retaining almost all of the [H^+^_SC_]. The small decrease in [H^+^_SC_] likely arises from Na being displaced from the sodalite sites by La and replacing supercage protons.^20,22,57^

**Figure 2:**
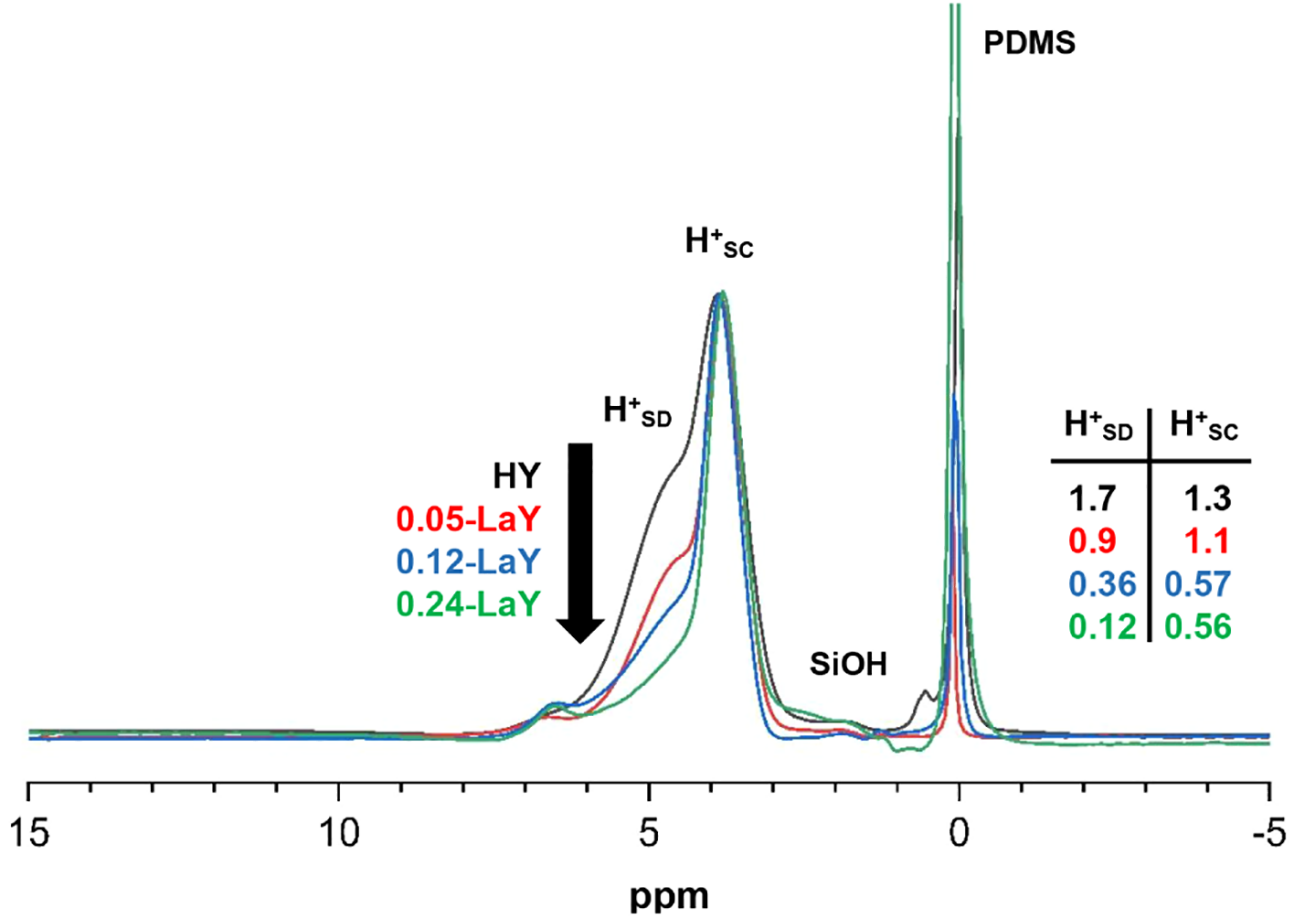
Overlay plot of ^1^H MAS NMR spectra for samples prepared from the parent NH_4_Y via low ramp-rate vacuum dehydration is described in Table 1, and arranged from top to bottom by the arrow and labeling on the left. The amount of H^+^_SD_ and H^+^_SC_ acid sites is given in the table on the right, in units of **mmol/g**. All spectra are normalized to the same height for the H^+^_SC_ peak at 3.9 ppm. Note the absence of any SiOH signals. For clarity, the PDMS peak is off-scale in some spectra.

Figure 1 shows that the experimental approach can quantitatively measure the impact of La incorporation on overall Brønsted acid site density and, more importantly, selectively quantify the location of La siting and its differential impact on sodalite vs supercage acid sites. The data in Figure 1 indicate that La preferentially resides in sodalite cages and, importantly, suggest that it exists as La^3+^ cations due to the absence of any new signals arising with La introduction that could be assigned to La^3–n^(OH)_n_ species. The overlay plot in Figure 2 confirms the findings from Figure 1 and further demonstrates that increasing the level of La introduction preferentially decreases the number of H^+^_SD_ sites. The two BAS peaks in Figure 2 are normalized to the same intensity for the H^+^_SC_ peak, which clearly shows that almost all sodalite BASs are eliminated by the time a 0.24 LaY loading is achieved, but supercage BASs remain. Again, no peaks are observed for SiOH species, and no peaks are observed that could be assigned to La^3-n^(OH)_n_ species even at the highest loading in Figure 2. Signals from La^3–n^(OH)_n_ species are observed for the 0.97, 2.4, and 4.8 LaY samples listed in Table 1*(vide infra)*.

### Preferred La Siting by Experiment and Theory

**Figure 3:**
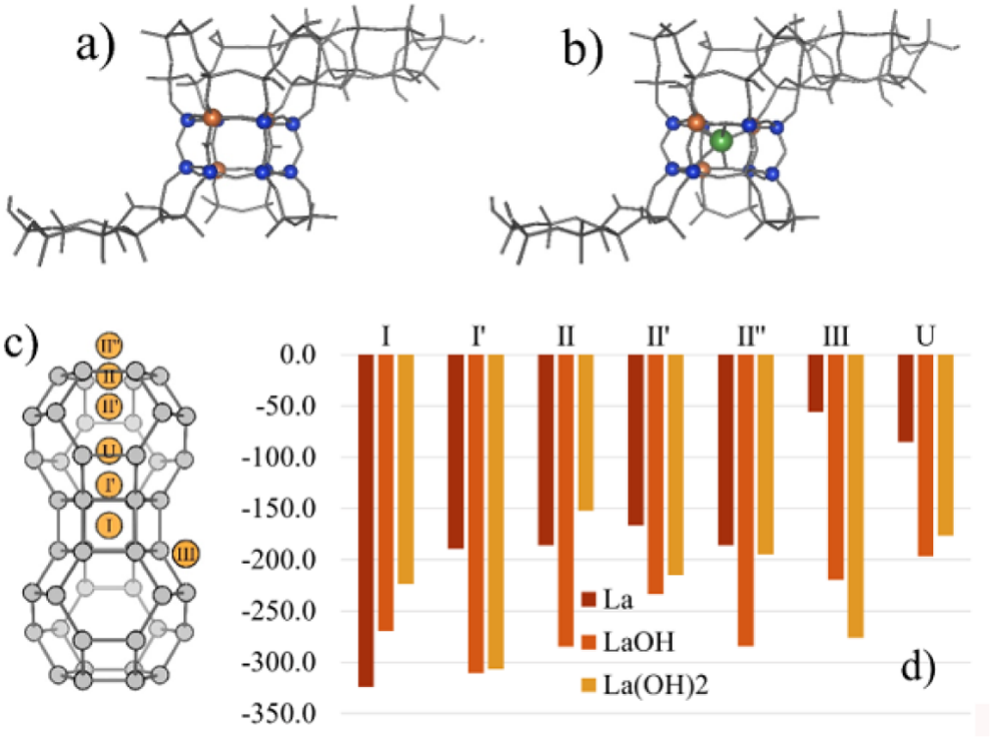
Portions of the DFT-calculated unit cell of zeolite Y (a) without La and (b) with La^3+^ in the hexagonal prism. (c) Schematic showing all potential cation locations, and (d) the energy in kJ/mol of a single La in each framework location as either (red) La^3+^, (orange) La(OH)^2+^, or (gold) La(OH)_2_^+^.

DFT calculations probing the expected fate of La-ion siting at low loadings are consistent with the experimental findings in Figures 1 and 2. The framework model had a total Si/Al ratio of 3 to agree with the experimental ^29^Si NMR results shown in Figures S2 and S3, requiring calculating arrangements of 12 Al atoms per unit cell. Three different Al distributions were possible following Loewenstein’s rule: 3 Al in each of the four hexagonal prisms (3333), 3 Al in two hexagonal prisms with the other two containing 2 and 4 Al atoms (4332), or all Al atoms in only two hexagonal prisms (6600). Predictably, the (6600) model was significantly less stable and can be disregarded, though the other two configurations were energetically similar. While the experimental ^29^Si NMR data were closer to the calculated spectrum of (4332) than (3333), as seen by comparing Figures S2 and S3, there are dissimilarities that indicate the catalyst is likely comprised of both modeled unit cells. Thus, the La location was determined for cages connected by a hexagonal prism with three Al atoms, as shown in Figure 3a. Within the faujasite framework, there are 7 unique positions in which La is potentially stable, as shown in the Figure 3c schematic. DFT calculations show that a single La ion is most stable as La^3+^ in site I within the hexagonal prism (Figure 3b). Since acid sites in the hexagonal prism are included in the H^+^_SD_ signal of ^1^H NMR spectra, and a cation in site I is most likely to titrate acid sites at I and I’ (in the sodalite cage),^58^ this corroborates the direct ^1^H NMR experimental data, which showed a preferential loss of sodalite acid sites. As La-loading increases, other less stable sites, in which La is more energetically stable as La(OH)^2+^ or La(OH)_2_^+^ are titrated. Even as less favorable sites are titrated, sodalite sites are still more energetically favorable than supercage sites, supporting the ^1^H NMR data, which shows that even as loading increases, sodalite acid sites are preferentially titrated. We also note that when entropic contributions were included in the calculations, the thermodynamics for the formation of highly dehydrated species are more favored due to the release of water molecules upon La-exchange. Similarly, isolated La species are also more favored than clustered La at low loadings, and the reader is directed to Figures S8 and S10 for the quantitative results.

**Figure 4:**
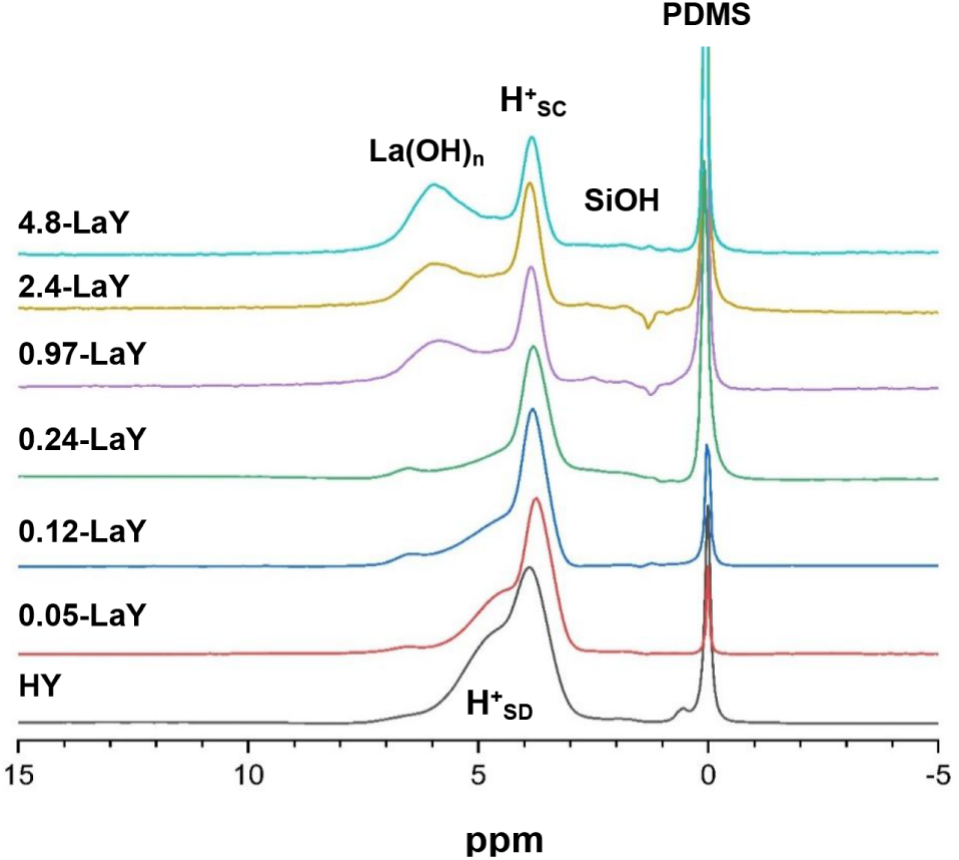
^1^H MAS NMR spectra for the entire suite of LaY catalysts listed in Table 1, showing the appearance of La(OH)_n_ signals at higher La loadings. Note the absence of any SiOH signals. PDMS denotes the internal standard polydimethylsiloxane. For clarity, the PDMS peak is off-scale in some spectra.

The data in Figures 1 and 2 coupled with the calculations summarized in Figure 3 indicate that LaY catalysts can be prepared in which La^3+^ resides in sodalite cage sites at the expense of BASs located there, and with no framework degradation corresponding to the formation of internal SiOH groups or La^3-n^(OH)_n_. Further, at sufficiently high La loadings, almost all H^+^_SD_ sites can be titrated, leaving a catalyst that essentially contains BASs only in the supercage. Figure 4 presents ^1^H MAS NMR data for the entire suite of LaY compositions listed in Table 1, demonstrating that at larger La-ion loadings, La(OH)_n_ species are observed, as indicated by the broad signal near 6 ppm for the 0.97-, 2.4-, and 4.8-LaY catalysts. Interestingly, even though increasing amounts of La(NO)_3_ were used in the synthesis solutions, the 0.97–4.8 LaY samples all have the same amount of La in the final catalysts, ca. 10 wt % or a La/Al ratio of 0.17, as shown in Table 1. LaY catalysts exhibiting the maximum La content that can be introduced using ion-exchange and heating methods, without causing any significant framework degradation, are the same catalysts that contain La(OH)_n_ moieties. For reference, Figure S4 shows the X-ray diffraction data, which, along with the ^29^Si NMR data previously shown in Figure S3, indicates the expected framework crystallinity. At high La loadings, some amorphous character is indicated by the broad background of the upper traces in Figure S4. Further, Figure S5 shows examples of ^1^H MAS NMR spectra for some of the same samples presented in Figures 1, 2, and 4 after controlled framework hydrolysis led to dealumination. In Figure S5, intense and clearly resolved signals in the 1.5–2.8 ppm region arising from SiOH and AlOH defect groups are observed, further supporting the conclusions stated above. With a well-characterized suite of defect-free variable-loading LaY catalysts, one can now interrogate the impact of controlled changes in the ratio of [H^+^_SD_]:[H^+^_SC_], the amount of La residing in the sodalite unit, and the presence of La(OH)_n_ species on catalyst performance.

### Impact of La Distribution on Catalyst Performance

Introducing lanthanum to zeolite Y is well known to improve the catalyst stability during hydrothermal regeneration steps, and some reports have also shown reactivity changes in specific reactions.^3,26,29,47,57^ Given that many prior reports employ catalysts with varying degrees of framework defects and residual Na cations, a direct assessment of the impact of La incorporation in sodalite vs supercage sites, as well as the impacts of La(OH)_n_ on catalyst performance, is lacking.

**Figure 5:**
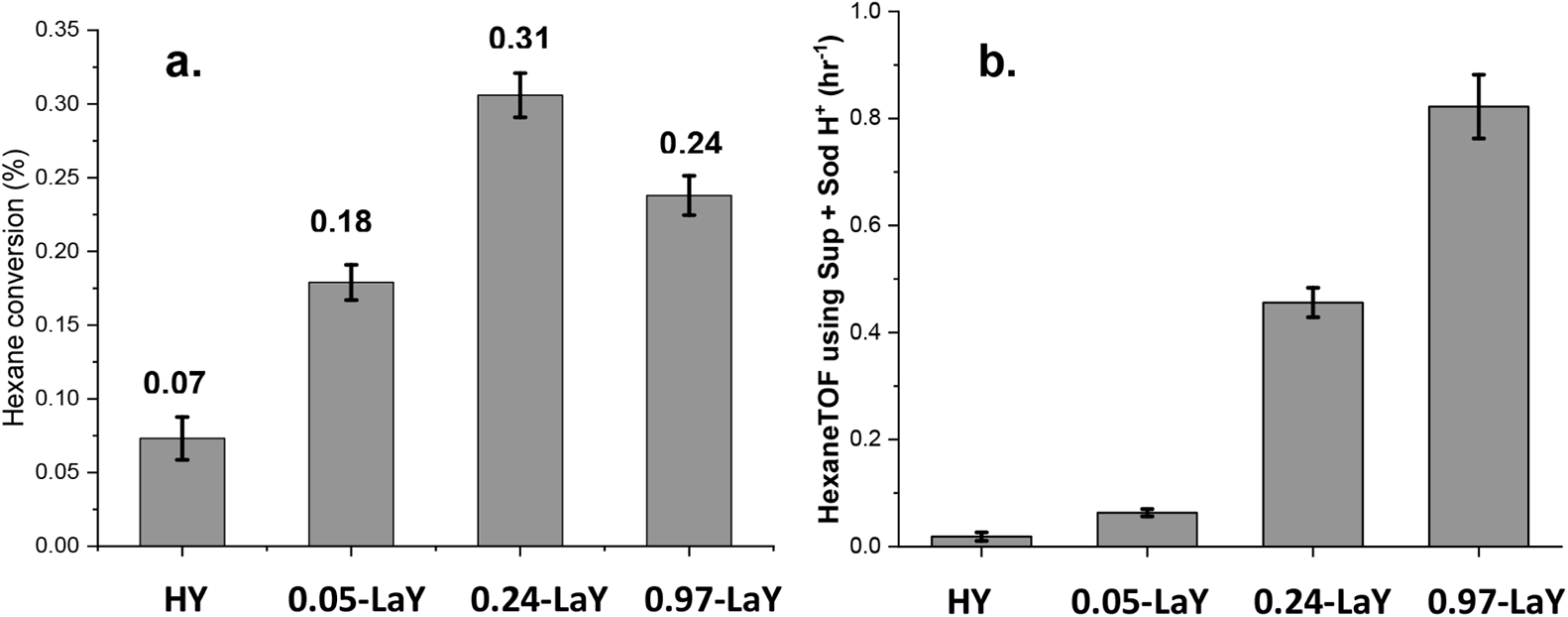
(a) Hexane conversion at 425 °C for HY and LaY catalysts with La content as indicated. Error bars represent the change in conversion during the 6 h time on stream in a flow reactor. (b) TOF was normalized to the sum of ([H^+^_SD_] + [H^+^_SC_]) BAS species. Importantly, additional plots of TOF based on the number of each type of BAS as well as the sum of all species ([H^+^_SD_] + [H^+^_SC_] + [La(OH)_n_]) are shown in Figure S7, with the latter demonstrating a decrease in TOF with detectable La(OH)_n_.

Figure 5a shows that the addition of only 0.04 La^3+^ per Al causes a significant increase in conversion for the flow-reactor hexane cracking reaction at 425 °C. The hexane conversion of the 0.05-LaY catalyst (i.e., 0.04 La per Al) is roughly double that of an HY sample of equivalent mass. Considering that the incorporation of La^3+^ cations results in a lower concentration of acid sites, the TOF of the catalyst effectively more than doubles, as shown in Figure 5b. Given that at least 80% of the La present in 0.05-LaY is located in the sodalite cages, this indicates that La^3+^ in the sodalite cages increases hydrocarbon cracking rates under typical gas-phase reaction conditions. Although an increase in conversion is also seen in 0.24-LaY compared to 0.05-LaY, it is not proportional to the increase in La content. The 0.24-LaY has roughly four times the La^3+^ content of 0.05-LaY, but its hexane conversion is not even double that of 0.05-LaY. Interestingly, increasing the lanthanum loading from 0.24-LaY to 0.97-LaY actually decreases the overall hexane conversion. A similar loss of activity at high La loadings has been reported in the literature, though the maximum La content varies significantly in literature reports, as do differing degrees of framework hydrolysis and internal defects.^7^ It is not clear why conversion decreases for the 0.97-LaY, although it is expected that with increasing La exchange for BAS protons, conversion will decrease. Indeed, Figure S7 shows TOF data as a function of all proton types and versus the sum ([H^+^_SD_] + [H^+^_SC_] + [La(OH)_n_]), clearly showing a decrease in TOF once lanthanum hydroxides are detected by the quantitative NMR data in Figures 1 and 2. From Figure 4, it was observed that the 0.97-La–Y sample contained La(OH)_n_ species and no clearly resolved H^+^_SD_ sites but fully resolved H^+^_SC_ sites. Indeed, because of the quantitative nature of the data in Figure 4 and fitting the three known species in the 3–6 ppm region to H^+^_SC_, H^+^_SD_, and La(OH)_n_ groups, the total number of BASs in the 0.97-LaY is 0.25 mmol/g, while the amount of La(OH)_n_ protons is 0.38 mmol/g. In each of the three high-La loading samples where the broad La(OH)_n_ peak at 6 ppm is observed, recalling the [H^+^_SD_] and [H^+^_SC_] values from the table on the right of Figure 2, it is clear that these amounts for the 0.97-LaY are much lower than for HY, 0.05-LaY, and 0.24-LaY, and thus the conversion decrease is somewhat expected. The clearly unexpected result is that the HY exhibits much lower conversion than *any* of the LaY’s, and most importantly, that introducing such a small amount of La at the expense of acidic protons in sodalite sites only, e.g., the 0.05-LaY, leads to a large increase in conversion. It is also important to note that, with respect to the data in Figure 5, preparation of LaY’s requires an additional aqueous exposure step that is not used in the HY catalyst, as the latter is prepared directly from deammoniation of the parent NH_4_Y. If the HY catalyst is itself exposed to liquid water, as is typical for an aqueous exchange sequence, then, as previously demonstrated, a significant decrease in conversion would be expected.^39^

To further address the unexpected results from the hexane cracking experiments and to identify the contributions of La in sodalite sites to reactivity, in situ isotopic-exchange MAS NMR experiments were conducted using toluene-d_8_ as the reagent. Rate constants for the reaction were determined via a least-squares fit of the growth of the toluene ^1^H NMR peak area, T(t), during the short-time initial-rate region of the room-temperature ^2^H/^1^H exchange curve using the equation previously given in the Experimental section, assuming first-order kinetics based on the initial excess of toluene-d_8_. Isotopic exchange was observed only for aromatic sites in the toluene and not at the methyl group, as no signals near 2 ppm were observed, as exemplified by the typical series of raw data shown in Figure S5. Isotopic exchange experiments have been reported for both toluene and benzene in zeolites, and for HY, it is commonly understood that these and other typical hydrocarbon reagents cannot access the sodalite BASs due to steric restrictions^47,59−63^ unless specific postsynthetic sodalite-cage-opening reagents are used.^64,65^ The in situ NMR experiments were conducted at room temperature, approximately 400° below the flow-reactor hexane cracking experiments, which affords the opportunity to assess whether La can potentially impact low-temperature processes and expands the range for identifying potential mechanisms by which both sodalite BASs and La improve reactivity.^61−64,66,67^

**Figure 6:**
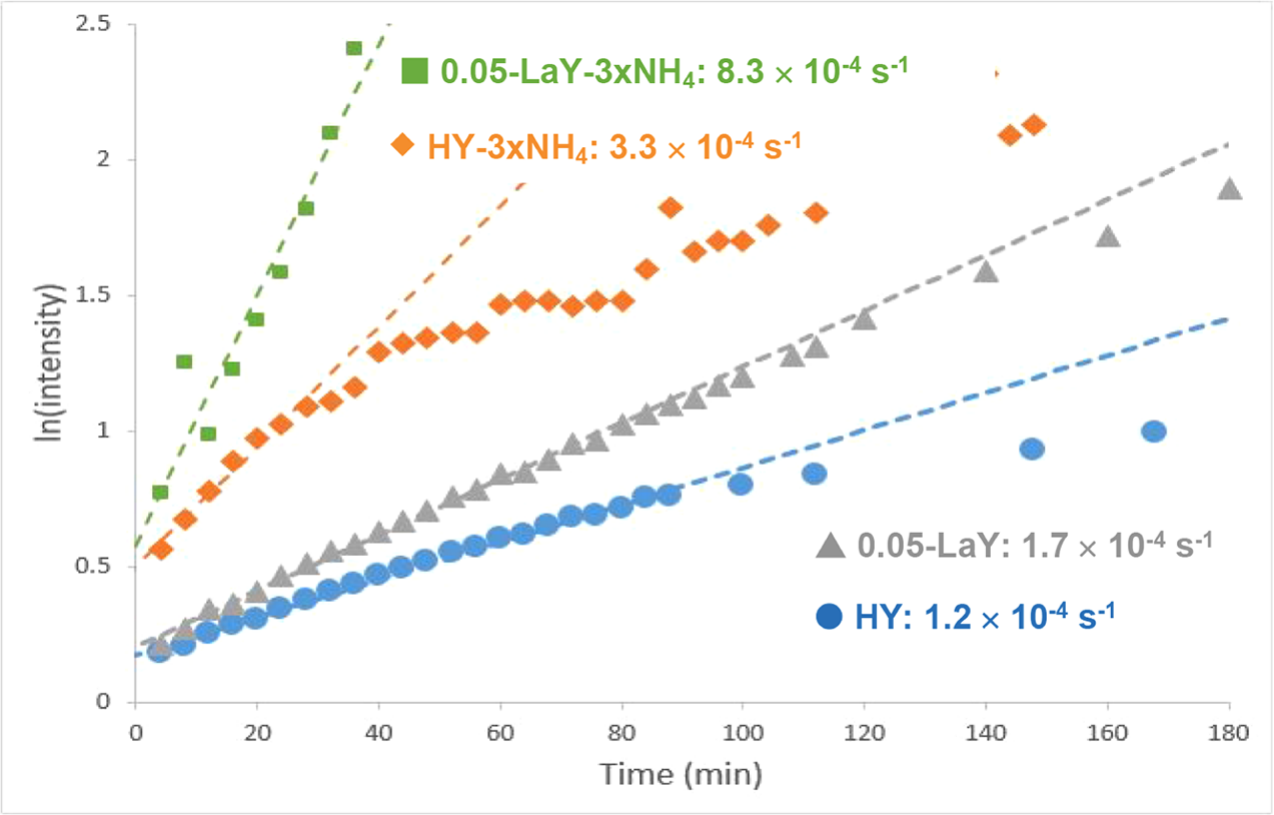
Kinetic results for room-temperature in situ MAS NMR experiments monitoring ^2^H/^1^H exchange between toluene-d_8_ and HY or LaY as denoted.

Figure 6 shows a semilogarithmic plot of the growth of the toluene peak area vs reaction time in the ^2^H/^1^H exchange experiment for toluene-d_8_ and the commercial or parent HY, and the 0.05-LaY samples. In addition, following the key findings shown in Figure 1 quantifying significant increases in [H^+^_SD_] following ammonium exchange of the parent HY, similar data for the three-times ammonium-exchanged HY (HY–3xNH_4_) and its comparable 0.05-LaY (0.05-LaY–3xNH_4_) version are presented. The four catalysts shown in Figure 6 are the same as those previously shown in Figure 1, and they are presented here to separate the impact of La displacing residual Na^+^ from La siting in the sodalite unit itself. The two lower data traces correspond to the parent HY and its 0.05-LaY variant, while the two upper data traces are from the HY–3xNH_4_ catalyst and its corresponding 0.05-LaY–3xNH_4_. Reaction rate constants reported in Figure 6 generally support findings from the hexane cracking data in Figure 5, in that incorporating even the smallest amount of La, with a concomitant decrease in the number of BASs according to the values for [H^+^_SD_] and [H^+^_SC_] presented in Figure 1*increases* the reaction rate constants. For the parent HY, La introduction leads to a 33% decrease in total [BAS] but increases the rate constant by 40%. Figure 6 shows that ammonium exchange of the parent HY, yielding the HY–3xNH_4_ catalyst, leads to almost a 3-fold increase in the rate constant. This increase must be attributed to the significant increase in [H^+^_SD_] (1.7→2.1 mmol/g) resulting from the removal of residual Na^+^, since Figure 1 showed that [H^+^_SC_] remained almost constant after ammonium exchange. Notably, a comparison of the HY and HY–3xNH_4_ results reveals that increasing the number of sodalite acid sites can have a large impact on reaction rates, even for a molecule that is ostensibly too large to access those sites.

Finally, Figure 6 shows that the introduction of La into the HY–3xNH_4_ catalyst, yielding 0.05-LaY–3xNH_4_ increases the reaction rate constant by a factor of 2.5 relative to that of its HY starting material. Again, as shown in the Figure 1 data, the 0.05-LaY–3xNH_4_ catalyst has almost 40% less H^+^_SD_ sites (1.3 vs 2.1 mmol/g) due to La exclusively displacing sodalite protons, but the same number of H^+^_SC_ sites (1.5 vs 1.5 mmol/g) as its parent HY, and yet it exhibits much higher reactivity. The high-temperature hexane cracking data and the room-temperature isotopic exchange results, interpreted with the benefit of the quantitative information provided by the spin-counting NMR method, reveal that the exclusive and preferred siting of La cations in sodalite sites leads to increased catalyst reactivity, even for molecules that are sterically precluded from such sites. Several postulates have been published to explain the indirect influence of sodalite acid sites on reactions,^61−67^ and the results shown here, with the additional benefit of quantitative site specificity and two different reaction types/temperatures, are commensurate with La cations perturbing electric field gradients within the catalyst.^62,68^ Further, Figure 5 shows that increasing amounts of La ultimately lead to a decrease in hexane-cracking catalyst performance, with a maximum conversion observed for the 0.24-LaY catalyst. While not shown in Figure 6, identical toluene ^2^H/^1^H exchange experiments were run for the 0.24- and 0.97-LaY’s, with the 0.24-LaY exhibiting the largest rate constant for any catalyst prepared from the parent HY at 3.3 × 10^–4^ s^–1^, in agreement with the trend shown in Figure 5 for hexane cracking. However, the 0.97-LaY showed an almost 3-fold decrease in rate constant compared to the 0.24-LaY, further demonstrating that as more La is introduced to the catalyst, the benefit of its incorporation into the preferred sodalite sites is ultimately negated by the continued decrease in the overall number of BASs. The La(OH)_n_ species present in the 0.97-LaY catalysts are not reactive in the toluene reaction, as their peak area remains constant throughout.

### Proposed Explanation of Increased Reactivity in the Presence of Low La Loadings

Many mechanisms have been proposed to explain increased reactivity due to cations in the sodalite cages, and the mechanism proposed here is not unique.^58−63^ However, the removal of residual sodium and the lack of framework defects provide additional support to one proposed mechanism in particular. The data collected on ^2^H/^1^H exchanges in HY and LaY show no indication of exchange taking place on the methyl group of toluene, since the only ^1^H signal for toluene at any point in the isotopic exchange arises on the aromatic ring. As the sodalite BASs are inaccessible to toluene except by the methyl group and have not been made accessible by framework hydrolysis, this precludes any direct exchange between toluene and sodalite BASs. Despite this, the increased reactivity of HY–3xNH_4_ in the important control experiment described in Figure 6, which gained acid sites primarily in the sodalite cages relative to the initial HY from which it was prepared, indicates that proximate H^+^_SD_ sites play a significant role in determining the reaction rate.

The addition of small amounts of La cations, essentially all of which are present in the sodalite unit, further increases reactivity. Indeed, La addition increases reactivity more effectively in the case of HY–3xNH_4_ than in HY. In contrast, higher loadings of La do not necessarily increase reactivity; for instance, the reaction rate of 0.97-LaY shows an almost 3-fold decrease relative to 0.24-LaY in the isotopic exchange studies. It is likely, then, that the mechanism by which La increases the reaction rates employs proximate H^+^_SD_ sites, and the lack of those sites in samples with excess La inhibits reactivity. This is consistent with the mechanism proposed for ^2^H/^1^H exchange with benzene by Almutairi and Hensen, in which the increased dipole moment of the transition state due to stretching of the O–H bond is stabilized by the cation in the sodalite cages—in this case, La^3+^—and its perturbation of the electric field gradients.^60,62^ Other studies by Hensen, Ryder, and others show that the H^+^_SD_ sites participate in hydrogen-transfer reactions by quantum tunneling; that is, by a proton moving from one oxygen site to another in the same AlO_4_ tetrahedron.^60,65−68^ Thus, while the addition of La^3+^ in the sodalite cages can stabilize the transition state by perturbing the electric field gradients, it does so most effectively when there are still H^+^_SD_ sites present to allow for proton hopping between sites. Therefore, we can propose that the synergy between proximate La and H^+^_SD_ is greater than that between proximate H^+^_SD_ sites themselves, which is also consistent with the hexane cracking TOF data in Figure 5.

### La Impact on HY Stability in the Presence of Ambient Moisture

HY undergoes both irreversible and reversible changes to the framework in the presence of liquid- and gas-phase water at elevated temperatures,^43,45^ and recently it has been shown that internal silanols and aluminols that are not associated with a BAS, as well as non-framework Al resulting from dealumination, form under routine room-temperature ion-exchange conditions.^39^Figure S6 shows ^1^H MAS NMR data for the same series of catalysts with and without La, before and after exposure to ambient humidity for 1 week. In all cases, characteristic peaks for SiOH and AlOH species arising from framework defects are observed following exposure, as indicated by the large signals in the 1.5–2.8 ppm region. These results are in contrast to what was previously reported in Figures 1, 2, and 4 in which defect-free HY and LaY were measured. ^27^Al NMR confirms the presence of a hexacoordinate Al species (not shown). Quantification of all species in spectra like those shown in Figure S6 for HY, 0.05-LaY, 0.24-LaY, 0.97-LaY, and 2.4-LaY reveals that more than half of the BASs are lost following exposure, indicating that the presence of La in the catalyst does not prevent water attack at framework Al sites for the La amounts incorporated here. Indeed, the 0.24-LaY, which exhibited the best performance in the hexane cracking and isotopic exchange reactions, loses more than half of its [BAS] after 1 week of moisture exposure, and the reaction rate constant for the H/D exchange experiment decreased from the pre-exposure value of 3.3 × 10^–4^s^–1^ to 0.67 × 10^–4^s^–1^ following exposure.

## Conclusions

A combination of spectroscopy and reactor experiments on HY and La-exchanged HY catalysts without hydrolysis defects, coupled with DFT computational results, reveals that La^3+^ cations preferentially reside inside sodalite cages and lead to significant increases in catalyst reactivity, even though the number of sodalite acid site protons decreases. Experiments and calculations indicate that lanthanum exists as La^3+^ in sodalite sites and not as hydroxylated moieties at the low La loadings revealed by the quantitative NMR experiments. This result is consistent with prior conclusions regarding cation siting in faujasites based on extensive X-ray analyses.^69^ The impact of La incorporation on sodalite vs supercage acid sites, as well as the introduction of La(OH)_n_ was quantified using spin-counting NMR experiments, which, coupled with high-temperature flow-reactor data and room-temperature isotopic exchange reactions, revealed an optimum La loading that, when exceeded, led to decreases in catalyst performance. At increased La loadings commensurate with the formation of La(OH)_n_ species, TOFs decrease in hexane cracking experiments, suggesting that “synergistic” effects from nonframework lanthanols are not responsible for increased reactivity. Rather, in total, the experimental and computational results suggest that proximate La^3+^ and H^+^_SD_ pairs lead to increased electric field gradients for polarizing reagents in excess of that afforded by proximate H^+^_SD_ pairs. In the absence of La incorporation, quantitative experiments on defect-free catalyst structures showed that small increases in the number of sodalite acid sites lead to large increases in catalyst reactivity, even for reagents that are too large to enter sodalite cages. In total, these findings indicate not only the preferred siting of La in sodalite cage positions of HY but also the active role of both proton and La sites in the sodalite cages, even for molecules that are ostensibly too large to access them. Further, the data provide quantitative guidance for determining optimum La amounts to employ in LaHY catalysts.

## Supporting Information Available

Additional information including XRD, DFT, and NMR data (PDF)
