# Supplementary material for: Experimental Detection of Preferred Lanthanum Siting in Zeolite Y and Its Impact on Catalyst Reactivity
Source: J Phys Chem C Nanomater Interfaces. 2025 Apr 23;129(18):8545–55. doi: 10.1021/acs.jpcc.5c00474 (PMC12067440; doi:10.1021/acs.jpcc.5c00474)
Supplement: Supplementary file 1 — jp5c00474_si_001.pdf [file jp5c00474_si_001.pdf]

Supporting Information

Experimental Detection of Preferred Lanthanum Siting in Zeolite Y and its Impact on Catalyst Reactivity

Anya Zornes, Omio Rani Das, Nabihan B. Abdul Rahman, Jacob Crouch, Steven Crossley, Bin Wang, Walter Alvarez, Matthew J. Wulfers, Daniel E. Resasco, and Jeffery L. White*

***Corresponding Author**

Jeffery L. White, 420 Engineering North, School of Chemical Engineering, Oklahoma State University, Stillwater, Oklahoma 74078, United States; email: ***jeff.white@okstate.edu***

Authors

Anya Zornes, 420 Engineering North, School of Chemical Engineering, Oklahoma State University, Stillwater, Oklahoma 74078, United States

Omio Rani Das, 420 Engineering North, School of Chemical Engineering, Oklahoma State University, Stillwater, Oklahoma 74078, United States

Nabihan B. Abdul Rahman, School of Chemical, Materials, and Biological Engineering, University of Oklahoma, Norman, Oklahoma 73019, United States

Jacob Crouch, School of Chemical, Materials, and Biological Engineering, University of Oklahoma, Norman, Oklahoma 73019, United States

Bin Wang, School of Chemical, Materials, and Biological Engineering, University of Oklahoma, Norman, Oklahoma 73019, United States

Steven Crossley, School of Chemical, Materials, and Biological Engineering, University of Oklahoma, Norman, Oklahoma 73019, United States

Daniel E. Resasco, School of Chemical, Materials, and Biological Engineering, University of Oklahoma, Norman, Oklahoma 73019, United States

Matthew J. Wulfers, Phillips 66, Bartlesville Technology Center, Bartlesville, Oklahoma 74004

Walter Alvarez, Phillips 66, Bartlesville Technology Center, Bartlesville, Oklahoma 74004

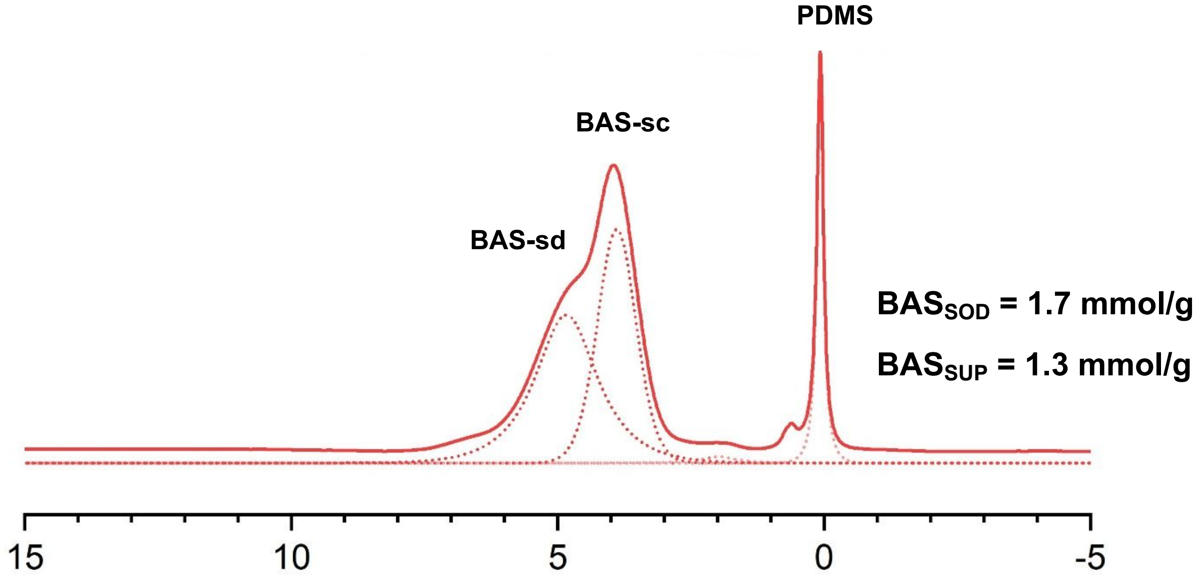

Figure S1. ^{1}H MAS NMR spectra of dehydrated and deammoniated zeolite HY, illustrating quantitative analysis of each type of BAS based on the PDMS standard addition.

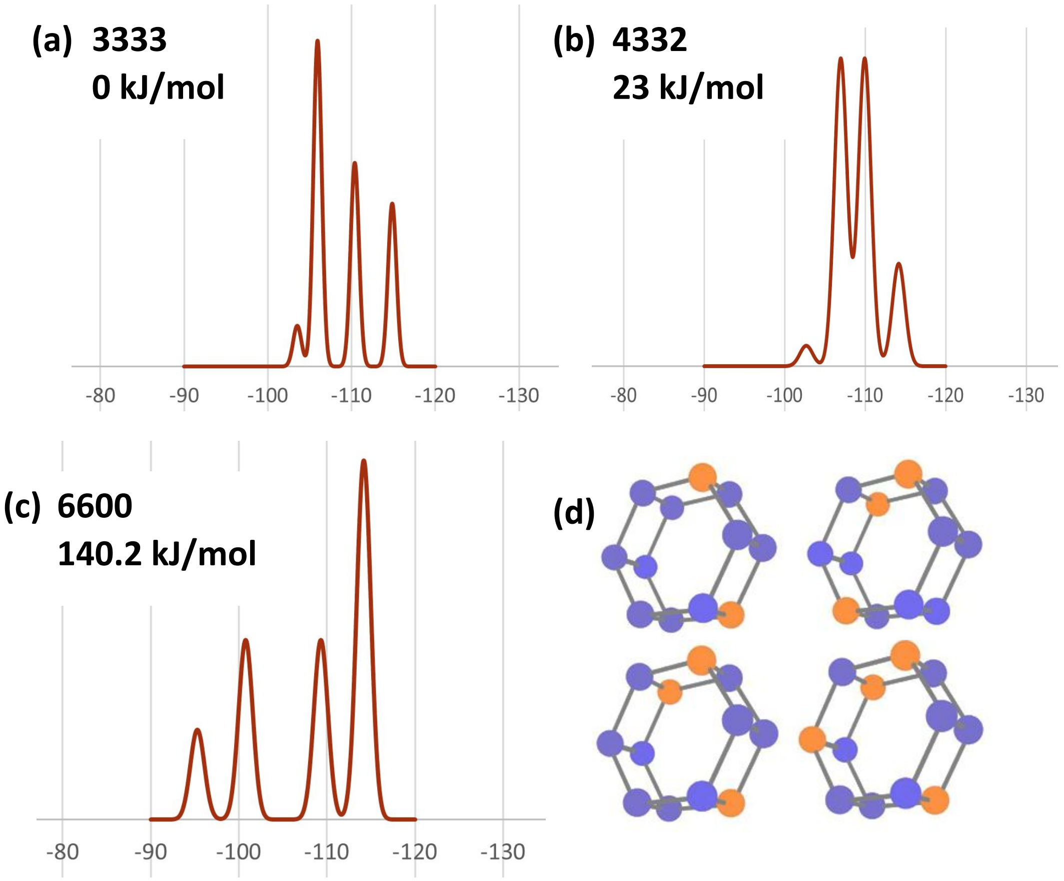

Figure S2. Calculated ^{29}Si NMR spectra of HY with three potential Al distributions (a) 3 Al in all four hexagonal prisms of a unit cell (3333), (b) 3 Al in two of the hexagonal prisms, 2 in one prism and 4 in the other (4332), and (c) 6 Al each in two of the hexagonal prisms with no Al in the other two. The most likely distribution is shown in (d), as experimental ^{29}Si NMR data on HY is closest to that of (b).

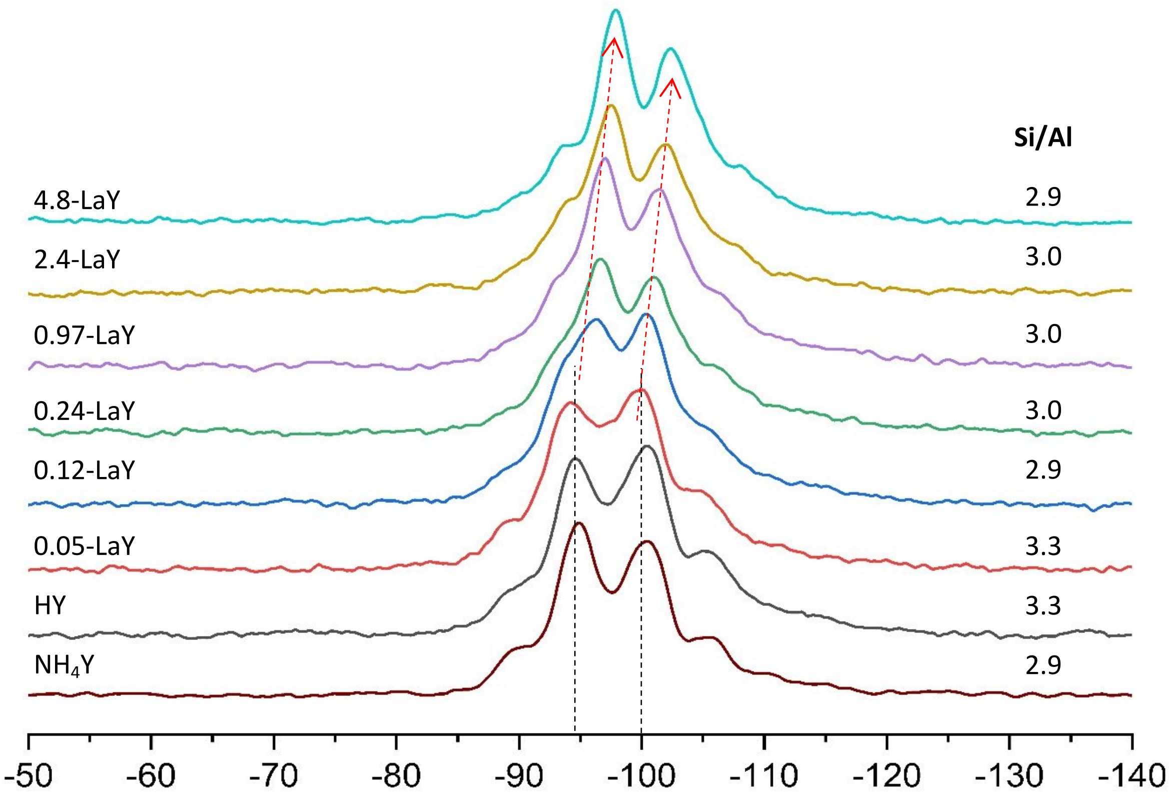

Figure S3. ^{29}Si MAS NMR of parent sample H-Y, and La-Y with varied La/Al ratios. H-Y was dehydrated to 450°C then rehydrated in ambient air, and La-Y samples were heated to 550°C then rehydrated in ambient air.

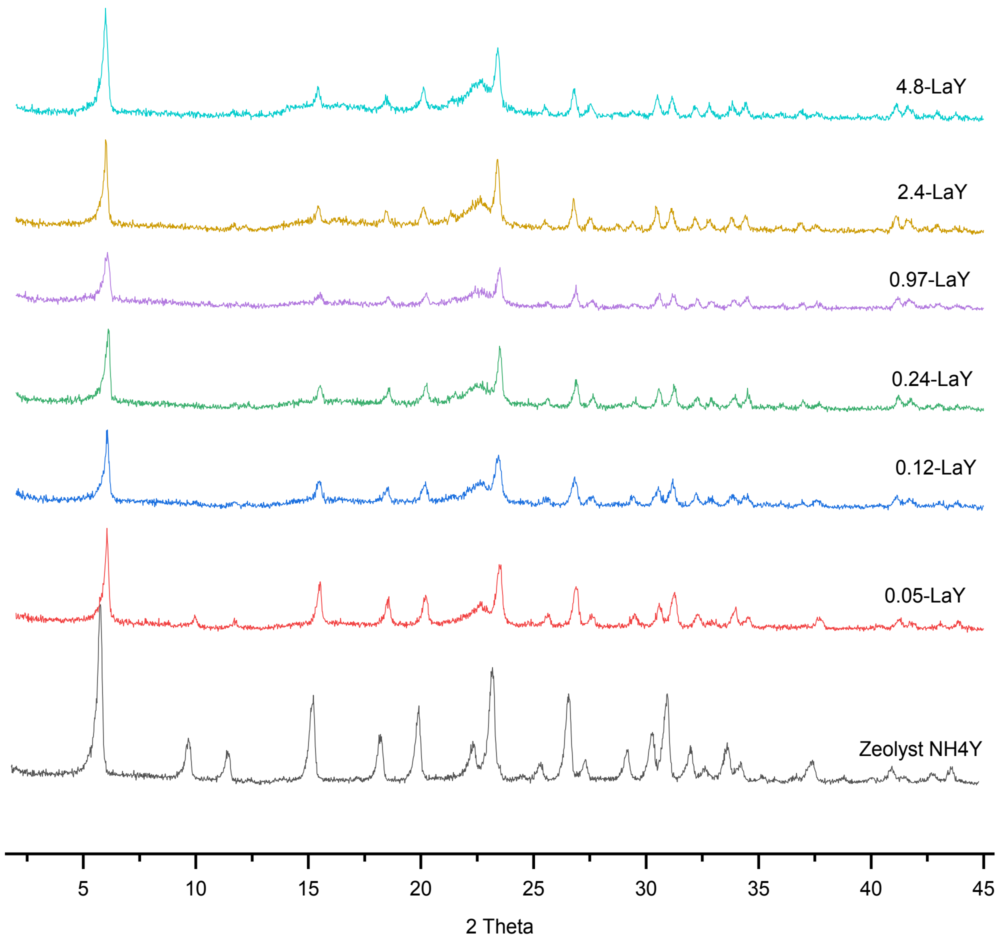

Figure S4. Powder x-ray diffractograms for the series of LaY catalysts listed in Table 1.

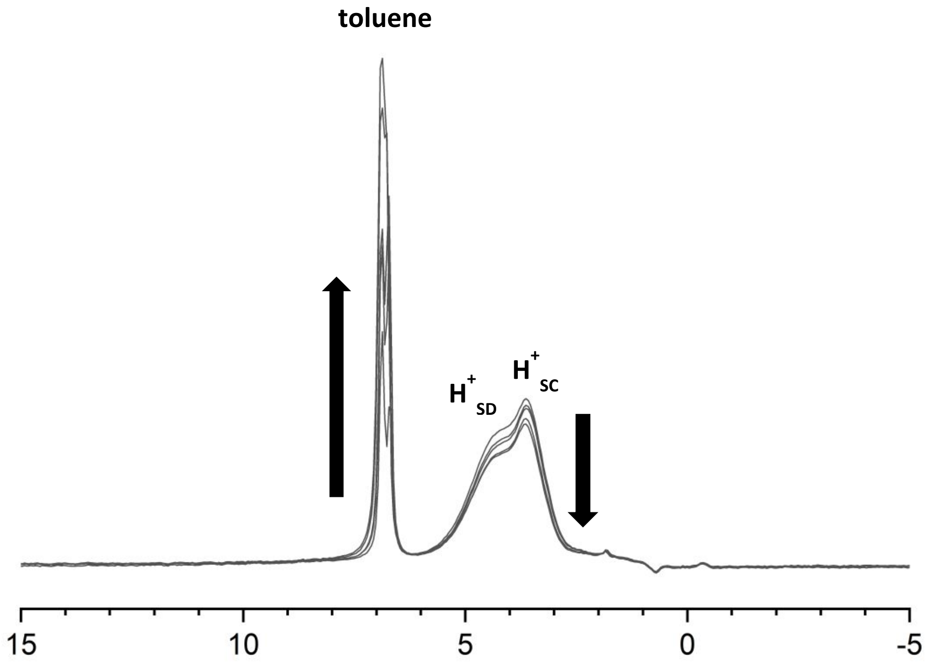

Figure S5. Example ^{1}H MAS NMR spectra following adsorption of toluene-d_8 onto an HY catalyst, as discussed in the text, showing increasing toluene signal near 7 ppm and decreasing BAS signals with increasing isotopic exchange time.

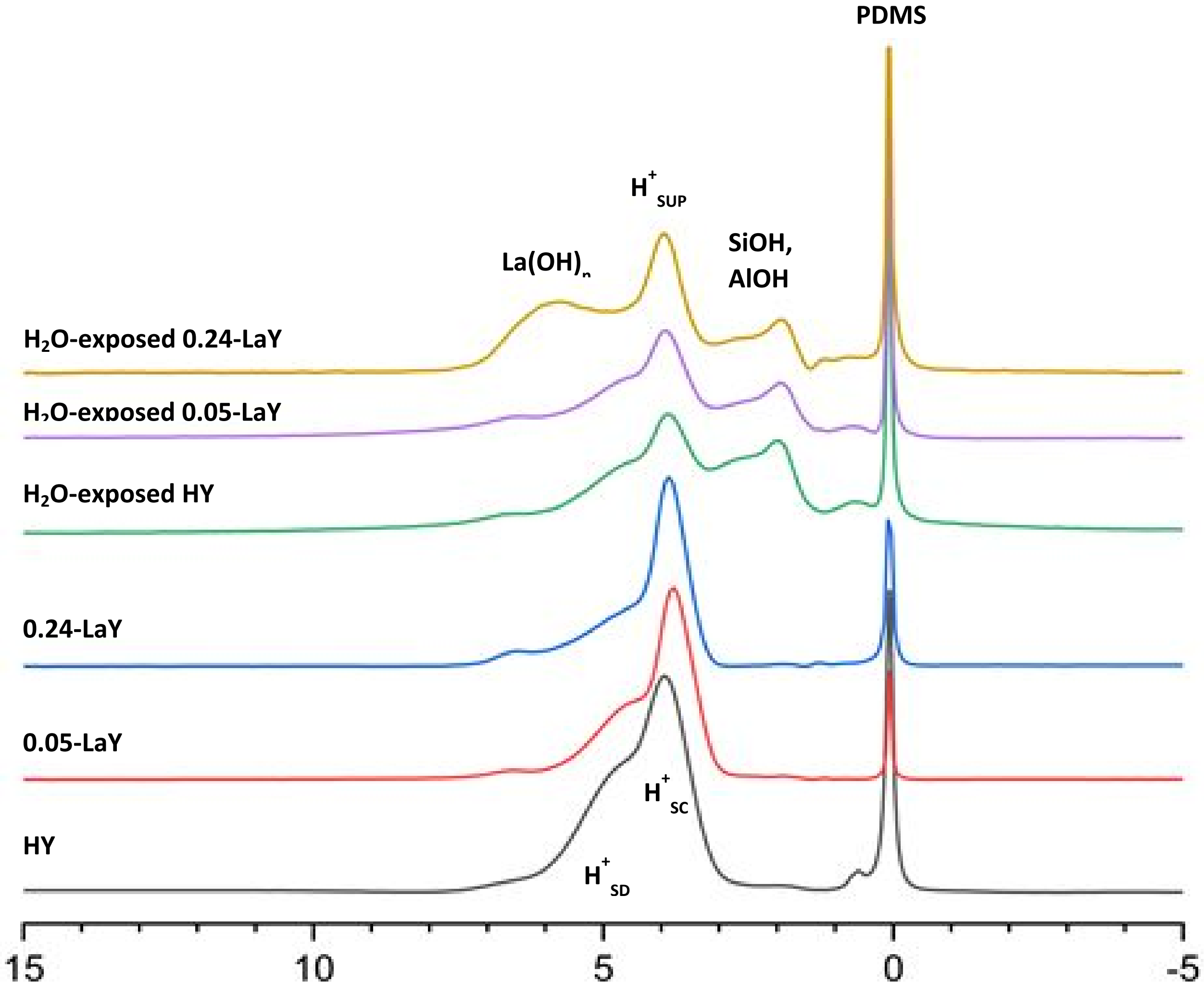

Figure S6. ^{1}H MAS NMR spectra of HY and LaY zeolites before and after controlled-humidity exposure and subsequent step-wise vacuum dehydration. Note the appearance of SiOH and AlOH signals in the 1.5-2.8 ppm region arising from framework hydrolysis, even as signals from BASs in sodalite and supercages are preserved, as well as the appearance of an $La(OH)_n$ signal in the dealuminated 0.24-LaY.

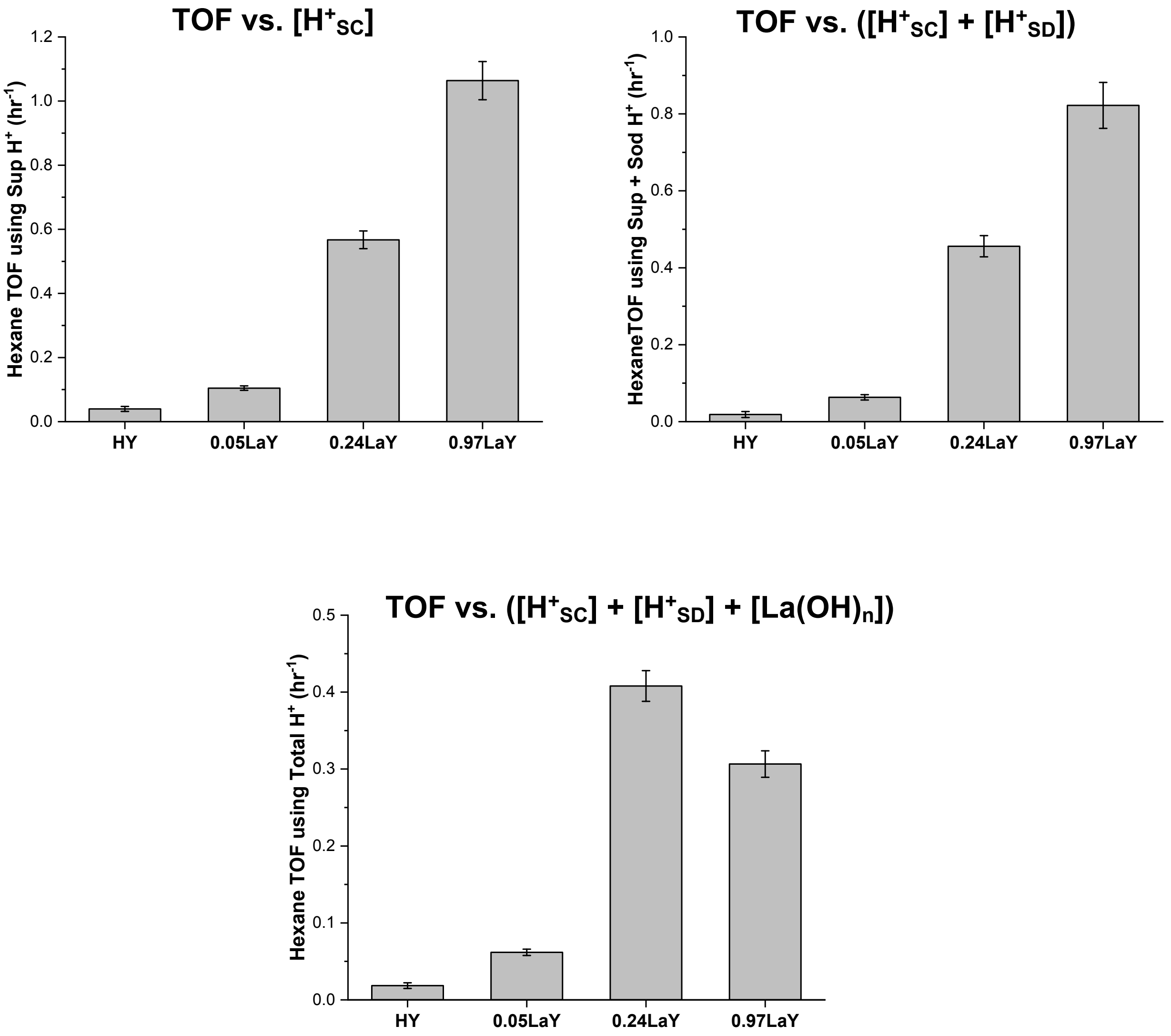

Figure S7. Turnover frequency (TOF) data for hexane conversion as a function of La loading as discussed in Figure 5. Data are normalized according to the number of sites as labeled in each figure.

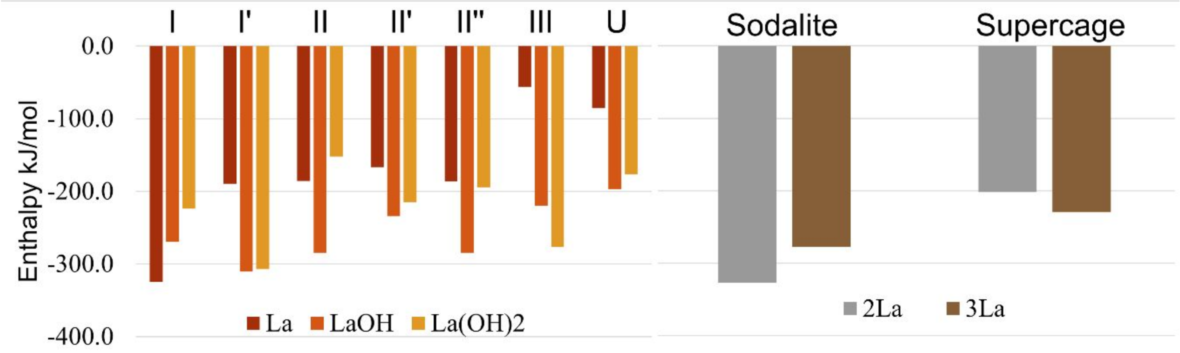

Figure S8. (Left) Enthalpy of lanthanum exchange for each hydroxide coordination at the seven unique sites. (Right) Enthalpy of lanthanum exchange for clusters of two and three inside either the sodalite or supercage. Values are reported on a per-lanthanum basis.

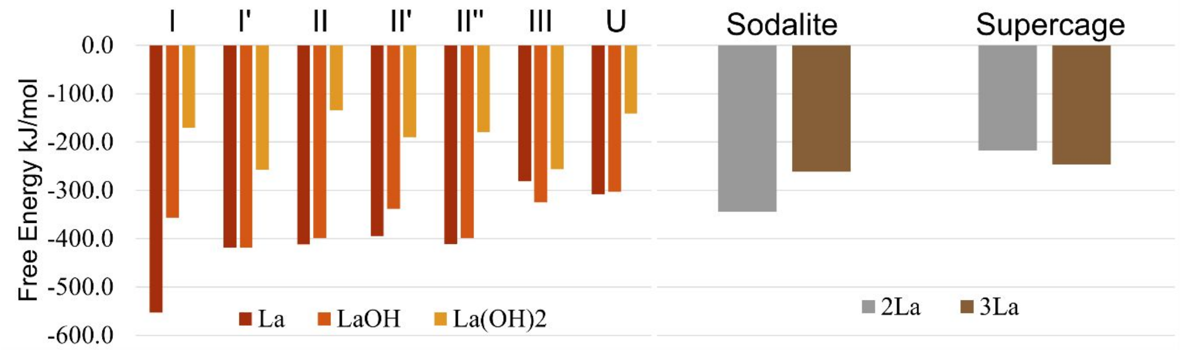

Figure S9. (Left) Gibbs Free Energy of lanthanum exchange for each hydroxide coordination at the seven unique sites. (Right) Gibbs Free Energy of lanthanum exchange for clusters of two and three inside either the sodalite or supercage. Gibbs Free Energy is calculated at 500 degrees Celsius. Values are reported on a per-lanthanum basis.

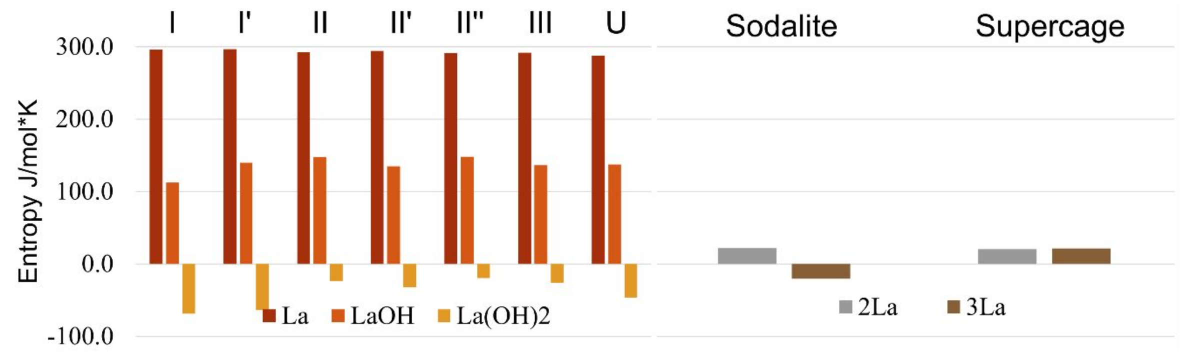

Figure S10. (Left) Entropy of lanthanum exchange for each hydroxide coordination at the seven unique sites. (Right) Entropy of lanthanum exchange for clusters of two and three inside either the sodalite or supercage. Values are reported on a per-lanthanum basis.

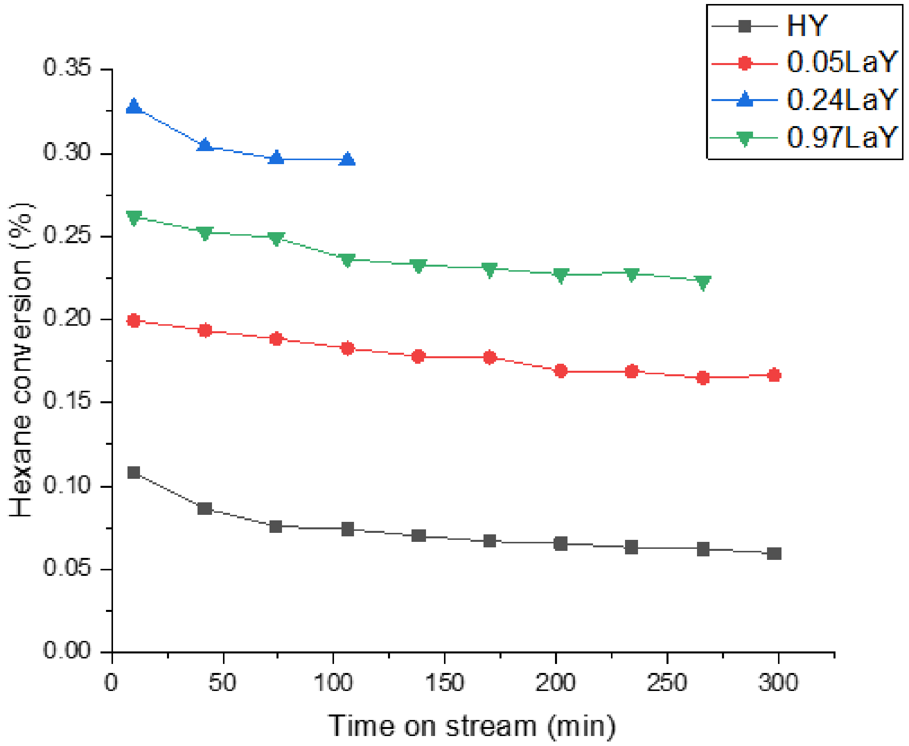

Figure S11. Hexane conversion as a function of time on stream during hexane cracking in a continuous-flow microreactor, where very mild catalyst deactivation is occurring but with similar changes in conversion for each catalyst. Reaction conditions: 50 mg catalyst, 0.5 mL/h liquid hexane injection rate (0.045 atm hexane partial pressure), 425°C, and 100 mL/min N_2 carrier gas flow.
